# Melatonin protects endothelial progenitor cells against AGE-induced apoptosis via autophagy flux stimulation and promotes wound healing in diabetic mice

**DOI:** 10.1038/s12276-018-0177-z

**Published:** 2018-11-21

**Authors:** Haiming Jin, Zengjie Zhang, Chengui Wang, Qian Tang, Jianle Wang, Xueqin Bai, Qingqing Wang, Majid Nisar, Naifeng Tian, Quan Wang, Cong Mao, Xiaolei Zhang, Xiangyang Wang

**Affiliations:** 1 Department of Orthopaedic Surgery, The Second Affiliated Hospital and Yuying Children’s Hospital of Wenzhou Medical University, Wenzhou, China; 2 Zhejiang Provincial Key Laboratory of Orthopaedics, Wenzhou, China; 3 Department of Radiology, First Affiliated Hospital of Wenzhou Medical University, Wenzhou, China; 4 Chinese Orthopaedic Regenerative Medicine Society, Hangzhou, China

## Abstract

Wound healing is delayed in diabetic patients. Increased apoptosis and endothelial progenitor cell (EPC) dysfunction are implicated in delayed diabetic wound healing. Melatonin, a major secretory product of the pineal gland, promotes diabetic wound healing; however, its mechanism of action remains unclear. Here, EPCs were isolated from the bone marrow of mice. Treatment of EPCs with melatonin alleviated advanced glycation end product (AGE)-induced apoptosis and cellular dysfunction. We further examined autophagy flux after melatonin treatment and found increased light chain 3 (LC3) and p62 protein levels in AGE-treated EPCs. However, lysosome-associated membrane protein 2 expression was decreased, indicating that autophagy flux was impaired in EPCs treated with AGEs. We then evaluated autophagy flux after melatonin treatment and found that melatonin increased the LC3 levels, but attenuated the accumulation of p62, suggesting a stimulatory effect of melatonin on autophagy flux. Blockage of autophagy flux by chloroquine partially abolished the protective effects of melatonin, indicating that autophagy flux is involved in the protective effects of melatonin. Furthermore, we found that the AMPK/mTOR signaling pathway is involved in autophagy flux stimulation by melatonin. An in vivo study also illustrated that melatonin treatment ameliorated impaired wound healing in a streptozotocin-induced diabetic wound healing model. Thus, our study shows that melatonin protects EPCs against apoptosis and dysfunction via autophagy flux stimulation and ameliorates impaired wound healing in vivo, providing insight into its mechanism of action in diabetic wound healing.

## Diabetes: Healing old wounds

Melatonin, a sleep-regulating hormone, may speed wound healing in patients with diabetes by protecting blood-borne wound-healing cells known as endothelial progenitor cells (EPCs). In diabetes, EPCs become damaged, lose their capacity to migrate to wounds and form new tissue, and die prematurely. Delayed healing can lead to ulcers, infection, and sometimes amputation. Melatonin has recently been reported to promote wound healing, but the mechanism remains unclear. Xiangyang Wang and Xiaolei Zhang at Wenzhou Medical University, China, and coworkers hypothesized that melatonin might protect EPCs from diabetes-induced damage. They found that melatonin improved EPCs’ ability to eliminate damaged components, allowing them to repair themselves and restoring their wound-healing function. In further experiments, diabetic mice treated with melatonin healed faster than untreated mice. These results may help improve treatments for complications of diabetes.

## Introduction

Diabetes, often referred to as diabetes mellitus, is a group of metabolic diseases in which blood glucose levels are elevated either because insulin production is inadequate or because the body’s cells do not respond properly to insulin, or both^1^. In 2014, it was estimated that over 422 million people throughout the world had diabetes^2^, and the number is predicted to increase. Most tissue impairment caused by diabetes is not due to the loss of glucose control but to complications of diabetes, including cardiovascular disease, nerve damage (neuropathy), kidney damage (nephropathy), eye damage (retinopathy), hearing impairment, and Alzheimer’s disease. Delayed wound healing is one of the most common diabetic complications^3^. The healing of surgical wounds^4^, dental extraction sockets^5^, and foot ulcers^6^ is retarded in diabetic patients. However, the pathogenesis of delayed wound healing caused by diabetes has not been fully elucidated; thus, effective treatments are still lacking.

There are four phases of the wound healing process:^7^ blood clotting (hemostasis), inflammation, tissue growth (proliferation), and tissue remodeling (maturation). Tissue growth is the phase most affected by diabetes during delayed wound healing, whereas angiogenesis is the beginning and the key step of the tissue growth phase. Endothelial progenitor cells (EPCs) are a population of cells that circulate in the blood^8^. In some situations, such as wound healing, they migrate to the injury site and differentiate into endothelial cells; therefore, they are considered of vital importance to vascular maintenance and angiogenesis^9^. Studies have reported that increased apoptosis and EPC dysfunction are implicated in delayed wound healing, including diabetes-induced delayed wound healing; therefore, EPCs are considered the primary target for wound healing therapeutics^10^.

Melatonin is a lipophilic molecule secreted by the pineal gland. It has been detected in almost all tissues, indicating its universal importance in the human body^11^. Studies have demonstrated the protective effects of melatonin during physiological and pathological conditions, including immune system regulation, cancer suppression, and anti-inflammation. Recent findings have suggested that melatonin promotes mucous epithelium healing^12^ and wound healing^13^. Melatonin also promotes diabetic wound healing in vitro;^14^ however, the mechanism of action of melatonin in diabetic wound healing remains unclear.

Macro-autophagy (here referred to as autophagy) is a lysosome-mediated intracellular catabolic mechanism responsible for the bulk degradation of damaged or dysfunctional cytoplasmic proteins and intracellular organelles; thus, autophagy may promote cell survival and homeostasis^15^. Autophagy is characterized by the engulfment of cellular components into double-membrane or multiple-membrane cytoplasmic vesicles called autophagosomes that form from a membranous structure called the phagophore. Autophagosomes ultimately fuse with lysosomes, forming autolysosomes, which ultimately degrade the engulfed proteins or organelles. This whole process is called autophagy flux. Autophagy dysfunction, especially autophagy flux impairment, has been implicated in the pathogenesis of many diseases, including vascular disorders; therefore, autophagy is considered an important target for disease therapy^16^.

Studies have shown that melatonin may modulate autophagy; however, this finding is controversial under different conditions. Kongsuphol et al.^17^ reported that melatonin suppresses autophagy in an SK-N-SH dopaminergic cell line, which was further supported by Yoo and Jeung^18^ in rat pituitary GH3 cells and by Chang et al.^19^ in mouse hippocampus in vivo. Other studies have suggested that melatonin induces autophagy, thereby protecting cells against apoptosis and senescence or the clearance of dysfunctional proteins^20,21^. Pre-treatment of EPCs with melatonin increased cellular renoprotective effects in a murine acute ischemic renal failure model;^22^ however, further studies are needed to determine whether melatonin modulates autophagy in EPCs and its function in autophagy flux in particular.

In the present study, we applied advanced glycation end products (AGEs), a common pathological mechanism in the apoptosis of EPCs in diabetic disorders, to induce oxidative stress. We investigated the effects of melatonin on apoptosis in EPCs under AGEs and melatonin treatment and its underlying mechanism of autophagy regulation. Further, we evaluated the therapeutic potential of melatonin in a streptozotocin (STZ)-induced diabetic wound healing model.

## Materials and methods

### Ethics statement

This study was approved by the Animal Care and Use Committee of Wenzhou Medical University. All surgical interventions, treatments, and postoperative animal care procedures were performed in accordance with the National Institutes of Health Guide for the Care and Use of Laboratory Animals.

### Chemicals

Ficoll-Paque PREMIUM was obtained from GE Healthcare (Buckinghamshire, UK). Melatonin, chloroquine (CQ), and dimethylsulfoxide (DMSO) were purchased from Sigma-Aldrich (St. Louis, MO, USA). Melatonin was dissolved in DMSO as a 200 mM stock solution and stored at −20 °C. Further dilution was performed in cell culture medium. Bovine serum albumin (BSA) and AGE-BSA (AGEs) were obtained from Merck-Millipore (Darmstadt, Germany). Compound C was purchased from Selleckchem (Houston, TX, USA). Primary antibodies against LC3, beclin-1, cleaved caspase-3, Bax, Bcl-2, caspase-9, and cytochrome c were obtained from Cell Signaling Technologies (Beverly, MA, USA). Primary antibodies against p-mTOR, mTOR, p-4EBP1, 4EBP1, p-AMPKα, AMPKα, p-p70S6K, p70S6K, and P62 and fluorescein isothiocyanate (FITC)-labeled and horseradish peroxidase-labeled secondary antibodies were purchased from Abcam (Cambridge, UK). Crystal violet and 4′,6-diamidino-2-phenylindole (DAPI) were obtained from Beyotime (Shanghai, China).

### EPC isolation and culture

EPCs were isolated from the bone marrow of mice. Briefly, mononuclear cells were separated from the tibia and femur of male ICR mice (6 weeks of age) using density gradient centrifugation. The cells were then cultured in a cell culture flask containing endothelial cell growth media (EGM-2) (Lonza, Basel, Switzerland). After 4 days of culture, non-adherent cells were removed by washing with phosphate-buffered saline (PBS), and adherent cells were incubated in fresh media for an additional 3 days. Cells from passages 3–5, namely, late EPCs, were used for the present study.

### EPC characterization

The morphology of EPCs in different growth periods was observed. Adherent cells were characterized by the uptake of Dil-acLDL (Biomedical Technologies, Stoughton, MA, USA) and FITC-labeled UEA-1 lectin (Vector Labs, Burlingame, CA, USA) staining. Cells were also characterized by co-immunofluorescence staining for CD31 and KDR expression. After staining, the samples were viewed under an inverted fluorescence microscope (Leica, Germany). The ability of passage 3–5 cells (late EPCs) to form capillary-like tubes in vitro was assessed on Matrigel.

### Cell treatment

To establish the apoptosis and dysfunction model of EPCs, different concentrations of AGEs (50, 100, 200, and 400 μg/mL) were added to the EPC culture medium for 24 h. Cells were pre-treated with different concentrations of melatonin (10, 20, 50, 100, and 200 μM) for 2 h before the addition of AGEs (400 μg/mL) to investigate its effect on cell apoptosis and dysfunction. To study the role of autophagy in melatonin-induced cell protection, EPCs were pre-treated with 10 μM CQ (an autophagy inhibitor) for 2 h prior to melatonin treatment. All experiments were performed in triplicate.

### Cell viability assay

Cell viability was assayed using the Cell Counting Kit-8 (Dojindo Co., Kumamoto, Japan) according to the manufacturer’s protocol. Passage 3–5 EPCs were plated onto 96-well plates (8000 cells/well) and incubated in EGM-2 at 37 °C for 24 h. The cells were then treated with BSA, AGEs, and melatonin as described above. After treatment, the cells were washed with PBS and 100 μL of non-fetal bovine serum (FBS) EGM-2 media containing 10 μL of Cell Counting Kit-8 solution were added to each well. The plate was then incubated for an additional 2 h. A microplate reader was used to measure the absorbance at 450 nm.

### Cell apoptosis analysis

The EPCs were treated with AGEs and melatonin as described above. Approximately 1 × 10^6^ cells were double-stained using the fluorescent dye annexin V-FITC/Propidium Iodide (PI) Apoptosis Detection Kit (Becton Dickinson, Franklin Lakes, NJ, USA) according to the manufacturer’s instructions. Apoptotic cells (annexin V^+^ /PI^−^) were detected using flow cytometry. The percentage of apoptotic cells was determined using the CytExpert software (Beckman Coulter, Brea, CA, USA).

### Cell migration assay

EPC migration was assessed using a modified Boyden chamber assay (Transwell, Coster, Cambridge, MA, USA). The EPCs were treated with AGEs and melatonin as described above and then detached using 0.25% trypsin. In total, 4 × 10^4^ cells in 200 μL non-FBS-containing EBM-2 were seeded into the upper chamber, while 700 μL of culture medium containing 2% FBS were placed in the lower chamber. After 24 h of incubation at 37 °C, the membrane was washed briefly with PBS and fixed with 4% paraformaldehyde. The membrane was then stained using hematoxylin solution, and the upper side of the membrane was wiped gently with cotton wool. Cells that had migrated to the lower side of the membrane were manually counted in every three random microscopic fields (×100).

### Tube formation assay

EPC tube formation was assessed using an in vitro Angiogenesis Assay Kit (Chemicon, Temecula, CA, USA). ECMatrix gel solution was mixed with ECMatrix diluent buffer and placed in a μ-Slide plate at 37 °C for 1 h to allow the matrix solution to solidify. The EPCs were pre-treated as described above and then harvested with trypsin/EDTA. In total, 2 × 10^4^ cells were placed into the wells containing the solid matrix with EGM-2 media at 37 °C for 16 h. Tube formation was observed using an inverted light microscope (×100). Three independent representative fields were assessed in each well, and the average numbers of tubes was determined.

### Cell matrix adhesion assay

EPC adhesion assays were performed as previously described. After pre-treatment as described above, EPCs were washed with PBS and then gently detached with trypsin/EDTA. Equal cell numbers were seeded onto fibronectin-coated 6-well plates and incubated for 30 min at 37 °C. After three gentle washes with PBS and subsequent fixation with 4% paraformaldehyde, adherent cells were detected by staining with DAPI. Images showing representative results from different independent experiments were acquired.

### Western blotting

Western blotting was performed using routine protocols. Treated cells were isolated using radioimmunoprecipitation assay buffer containing 1 mM phenylmethanesulfonyl fluoride, and protein concentration was measured using a Bicinchoninic Acid Protein Assay Kit (Beyotime). Protein samples (30 μg) were separated on a 12% sodium dodecyl sulfate-polyacrylamide gel, transferred to a nitrocellulose membrane (Life Technologies, Gaithersburg, MD, USA), and blocked. The membranes were incubated overnight at 4 °C with one of the following primary antibodies: LC3, beclin-1, cleaved caspase-3, Bax, Bcl-2, caspase-9, cytochrome c, p-mTOR, mTOR, p-4EBP1, 4EBP1, p-AMPKα, AMPKα, p-p70S6K, p70S6K, or P62. The densities of the protein bands were detected using the ChemiDicTM XRS + Imaging System (Bio-Rad, Hercules, CA, USA). Densitometric quantification of the membranes was performed using ImageJ.

### mPTP opening

Treated cells were collected and incubated with calcein-AM/cobalt to determine the function of the mitochondrial permeability transition pore (mPTP) protein. Calcein-AM causes diffuse calcein fluorescence throughout the entire cell, whereas cobalt quenches cytoplasmic calcein. The stimulation can lead to the opening of mPTP, which results in the translocation of cobalt from the cytoplasm to mitochondria. The presence of cobalt in the mitochondria quenches mitochondrial calcein. Thus, the calcein fluorescence intensity level can be used as a readout for the loss of mitochondrial integrity or mPTP function.

### MMP assay

MitoTracker Red CMXRos (Molecular Probes™, Thermo Fisher Scientific Inc., Waltham, MA, USA), a red fluorescent dye whose accumulation is dependent on membrane potential, was used to measure mitochondrial fission. Pre-treated EPCs were stained at a final concentration of 50 nM for 30 min at 37 °C, and nuclei were stained with Hoechst 33258 dye. Three random microscopic fields were taken per slide using a fluorescence microscope (Olympus Inc., Tokyo, Japan), and the fluorescence intensity was measured using ImageJ.

### Immunofluorescence

The EPCs were plated onto slides from a 6-well plate and then treated as described above. Paraformaldehyde (4%) was used to fix cells for 15 min at room temperature. After three washes with PBS, the cells were permeabilized for 5 min using 0.25% Triton X-100. After blocking with 10% FBS, the cells were incubated with the primary antibody (1:200) overnight at 4°C. The next day, the slides were washed and incubated with secondary antibodies for 1 h and labeled with DAPI for 1 min. Finally, a fluorescence microscope (Olympus Inc.) was used to examine three randomly chosen fields from each slide for microscopic observation.

### STZ-induced diabetes

Seven-week-old male ICR mice were purchased from the Animal Center of the Chinese Academy of Sciences in Shanghai, China. All ICR mice were housed in a specific pathogen-free room with a 12-h light/dark cycle and provided regular food and water for 1 week prior to any experimental procedures. Next, 116 ICR mice were randomly divided into two groups: normal (*n* = 48) and diabetes (*n* = 58). Diabetes was induced using a single intraperitoneal injection of 100 mg/kg STZ (Sigma-Aldrich) in citrate buffer (pH 4.5). Hyperglycemia was confirmed if the glucose level exceeded 250 mg/dL in heparinized tail vein blood (measured by a glucometer) 2 weeks after treatment with STZ. The mice with a blood glucose level above 300 mg/dL were considered diabetic and used in our experiment. Blood glucose and body weight were measured for all animals before injection and 7 and 14 days after injection. Ten mice in the diabetes group died within 14 days of injection.

### In vivo wound healing model and drug administration

Forty-eight non-diabetic (control) mice were randomly divided into either the control group (*n* = 24) or the control + melatonin group (*n* = 24). The diabetic mice were randomly divided into either the diabetes group (*n* = 24) or the diabetes + melatonin group (*n* = 24). All mice were subjected to induced surgery 2 weeks after STZ injection. The mice were anesthetized using 0.6% pentobarbital sodium (40 mg/kg) and the dorsal area was shaved. Next, two round, full-thickness dermal wounds of 0.385 cm^2^ (0.7 cm diameter) were made on both sides of the dorsal trunk using fine scissors, and 0.5-mm-thick silicone, donut-shaped splints (external diameter 13 mm, internal diameter 8 mm) were fixed on either side of the dorsal midline using 5-0 Prolene sutures. Melatonin was dissolved in DMSO at 50 mg/mL and then diluted with normal saline. Mice in the control + melatonin and diabetes + melatonin groups were treated with an intraperitoneal injection of melatonin solution at a dose of 10 mg/kg per day from the day of surgery until the mice were sacrificed, whereas mice in the remaining groups were administered an equivalent volume of saline. At the end of 3, 7, 14, and 21 days of wounding, mice from each group (*n* = 6) were sacrificed under pentobarbital sodium anesthesia, and tissue surrounding wounds were harvested for histological evaluation.

### Histological analysis

Skin tissues were fixed with 4% paraformaldehyde overnight and embedded in paraffin. The tissues were cut into 4-μm sections for routine hematoxylin and eosin (H&E) staining and collagen formation by Masson’s trichrome staining (Beyotime). Sections were analyzed, and images were captured with an optical microscope.

### Tissue immunohistochemical staining

Day 7 tissue sections were obtained as described above and heated for antigen recovery in 10 mM sodium citrate buffer (pH 6.0). After washing, the samples were blocked using 10% FBS for 30 min at room temperature. The tissue sections were stained with rabbit α-smooth muscle actin (α-SMA) antibody followed by Texas red-conjugated anti-IgG secondary antibody, and nuclei were stained with DAPI. All fluorescent images were taken using a fluorescence microscope (Olympus Inc.).

### Statistical analysis

The experiments were performed at least three times. Data are expressed as the mean ± standard error of the mean (SEM). Statistical analysis was performed using GraphPad Prism Version 5.0 software (GraphPad Software, San Diego, CA, USA). Inter-group comparisons were performed using one-way analysis of variance followed by Tukey’s test. Probability values of *p* < 0.05 were considered statistically significant.

## Results

### Morphology and characterization of bone marrow-derived EPCs

Proliferative endothelial colonies were observed at 3–7 days during EPC culture, and a “cobblestone-like” morphology typical of mature EPCs was observed at 7–14 days (Fig. S1A). EPCs were identified as double-positive for Dil-acLDL uptake and FITC-labeled UEA-1 lectin-binding affinity. More than 90% of EPCs stained positive for acLDL (red) and UEA-1 (green) (Fig. S1B). To further confirm the identity of these cells, immunofluorescence staining was performed to analyze the expression of specific cell markers. The results illustrated the expression of endothelial markers (CD31 and KDR) in the majority of EPCs (Fig. S1C). As shown in Figure S1D, angiogenesis of late EPCs was also observed, and late EPCs successfully formed tubulin-like structures on Matrigel.

### Melatonin treatment decreases AGE-induced apoptosis in EPCs

**Fig. 1:**
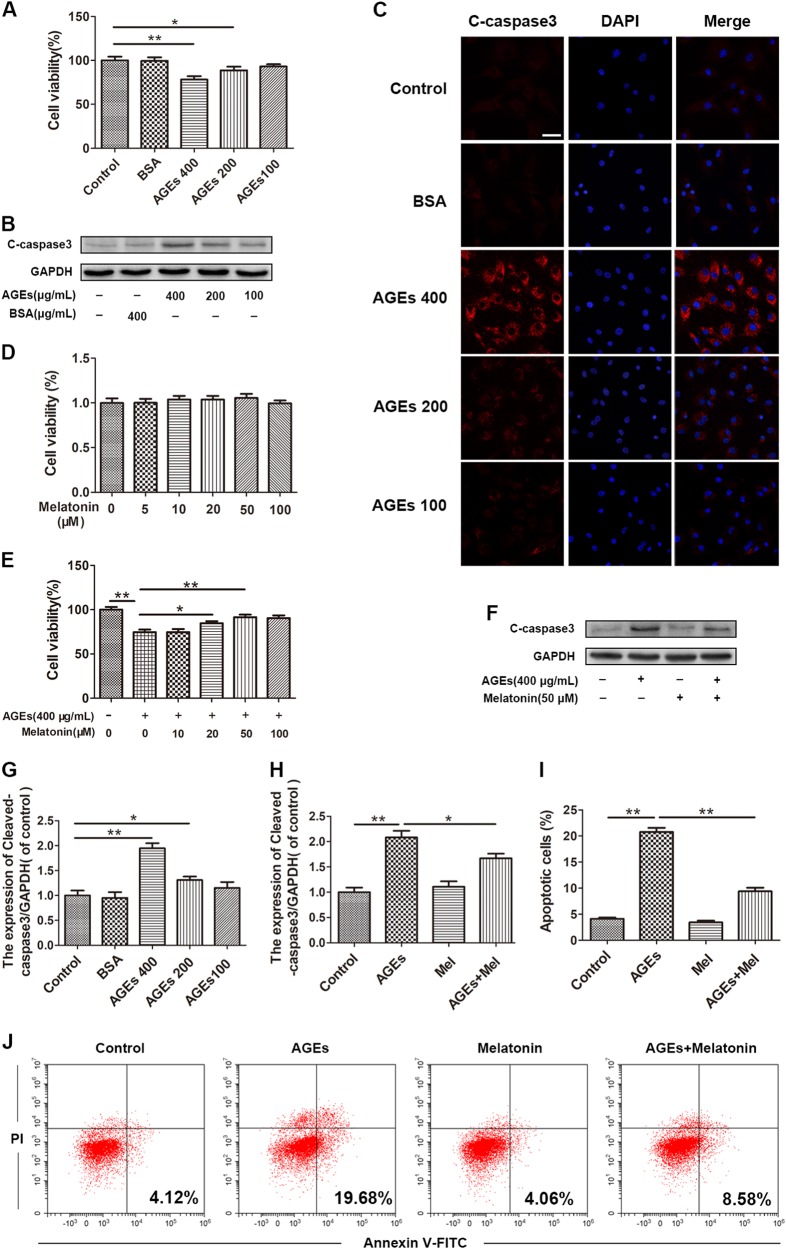
Melatonin treatment decreases AGE-induced apoptosis in EPCs. **a** Cell Counting Kit-8 results of EPCs treated with BSA and different concentrations of AGEs for 24 h. **b**–**c**, **g** Immunofluorescence and Western blot analysis of the protein expression levels of cleaved caspase-3 in treated EPCs. Scale bar, 25 μm. **d** Cell Counting Kit-8 results of EPCs treated with different concentrations of melatonin for 24 h. **e** Cell Counting Kit-8 results of melatonin-pre-treated EPCs induced by AGEs. **f**, **h** Protein content of cleaved caspase-3 in EPCs treated with AGEs and melatonin. **i**, **j** Apoptosis was detected using the annexin V-FITC/PI kit. Typical images of apoptosis of different groups are shown, as measured by the CytExpert software. Data are presented as the mean ± SEM. Significant differences between the treatment and control groups are indicated as **P < 0.01 or *P < 0.05. *n* = 3

As shown in Fig. 1a, the cell viability of EPCs decreased following AGEs treatment in a dose-dependent manner and, at a concentration of 400 μg/mL, a 22% decrease in cell viability was observed. Compared to the control group, invariant cell viability was observed following BSA treatment, indicating that BSA is not cytotoxic to EPCs. The Cell Counting Kit-8 assay also showed that melatonin was not cytotoxic to EPCs at concentrations of ≤100 μM after 24 h of treatment and had a protective effect against AGE-induced cell death (Fig. 1d, e). Western blotting and immunofluorescence results showed that AGEs markedly increased the expression of the apoptosis-related protein cleaved caspase-3 (Fig. 1b, c, g), whereas pre-treatment with 50 μM melatonin for 4 h inhibited elevated cleaved caspase-3 protein expression induced by AGEs (Fig. 1f, h). The cell apoptosis analysis also showed that AGEs induced a greater degree of apoptosis, whereas pre-treatment with melatonin significantly attenuated it (Fig. 1i, j). These results indicate that melatonin decreases AGE-induced apoptosis in EPCs.

### Melatonin alleviates AGE-induced apoptosis by reducing mitochondrial functional damage

**Fig. 2:**
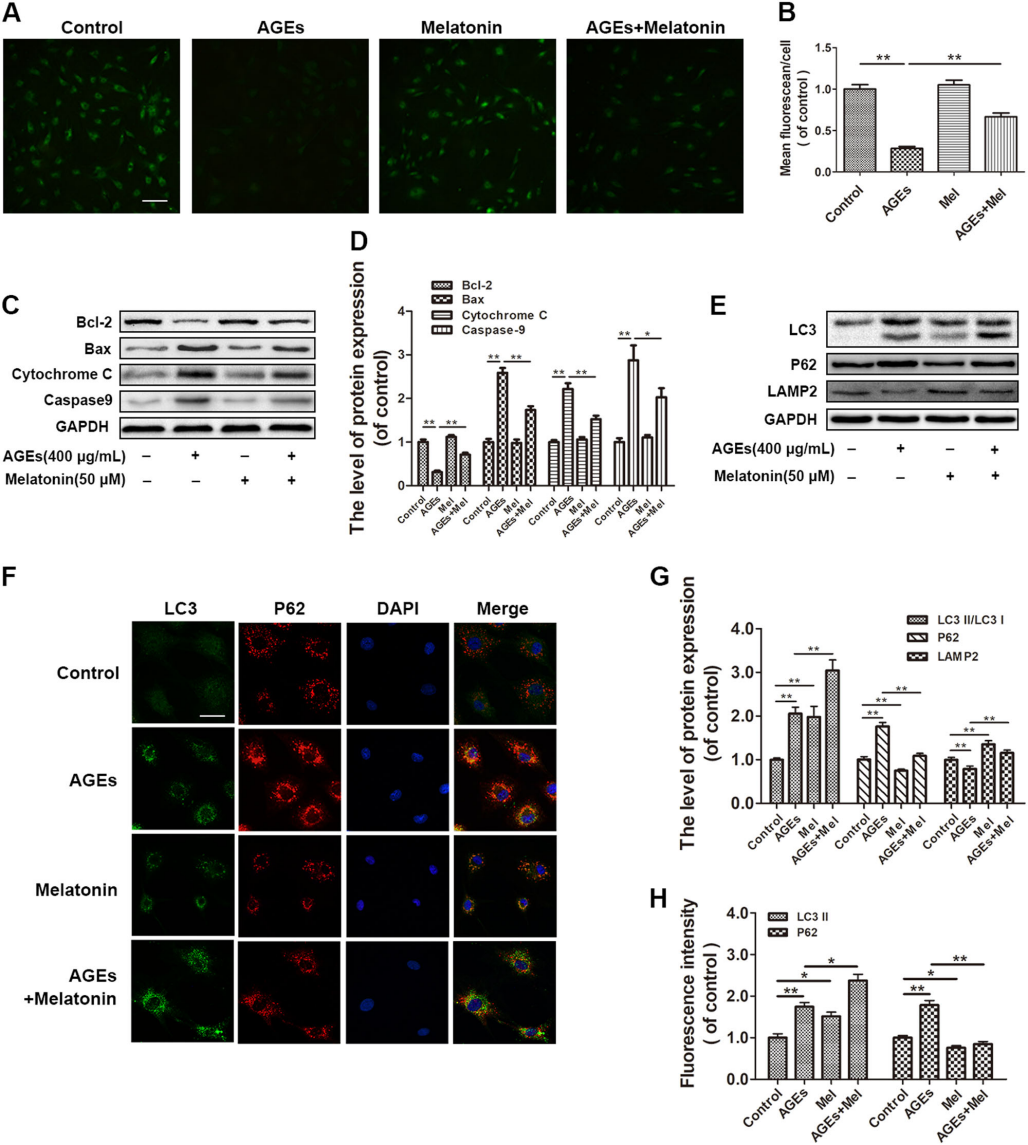
Melatonin reduces mitochondrial functional damage and promotes autophagy flux in AGE-treated EPCs. EPCs were pre-treated with 50 μM melatonin for 2 h and then 400 μg/mL AGEs were added for an additional 24 h. **a**, **b** Mitochondrial permeability transition pore (mPTP) opening was detected by co-loading with calcein-AM and cobalt. Scale bar, 50 μm. **c**, **d** Protein expression levels of Bcl-2, Bax, cytochrome c, and caspase-9 in EPCs of each group treated as described above. **e**, **g** Protein expression levels of LC3, p62, and LAMP2 in EPCs treated as described above. **f**, **h** Double immunofluorescence of LC3 protein (green) and p62 protein (red) in EPCs. Scale bar, 25 μm. Data are presented as the mean ± SEM. Significant differences between the treatment and control groups are indicated as **P < 0.01 or *P < 0.05. *n* = 3

As mitochondrial function is closely related to cellular apoptosis, we used the calcein-AM/cobalt quenching method to examine the effects of melatonin on mitochondrial function in EPCs. As shown in Fig. 2a, b, AGE stimulation resulted in a 2.4-fold reduction (*P* < 0.05) in mitochondrial green fluorescence in cells, suggesting that the AGEs influenced mitochondrial integrity, leading to the translocation of cobalt from the cytoplasm to mitochondria. Importantly, the AGE-induced reduction of fluorescence was attenuated by melatonin treatment, which indicates that the mPTP opening was reduced. Next, we assessed the expression of apoptotic proteins related to mitochondrial function: Bcl-2, Bax, caspase-9, and cytochrome c. The expression of Bcl-2 in the AGE-treated group was downregulated, while Bax, caspase-9, and cytochrome c expression was upregulated (Fig. 2c). However, after melatonin treatment, Bcl-2 was upregulated by 126% (*P* < 0.01) compared with the AGE-treated group. In contrast, Bax, caspase-9, and cytochrome c expression decreased to 63% (*P* *<* 0.01), 70% (*P* < 0.01), and 68% (*P* < 0.01), respectively, relative to the expression levels in the AGE-treated group (Fig. 2d). Taken together, these data indicate that melatonin prevents AGE-induced mitochondrial dysfunction in EPCs.

### Melatonin promotes autophagy flux in AGEs-treated EPCs

The dynamic process of autophagy consists of two aspects: the formation and degradation of autophagosomes. Microtubule-associated protein light chain 3 (LC3) is a marker for autophagy, and the LC3-II/LC3-I ratio correlates with the number of autophagosomes^23^. In this study, immunofluorescence and Western blotting were used to assess autophagy protein LC3 levels. Immunofluorescence results showed that LC3-II fluorescence intensity increased in EPCs following treatment with AGEs or melatonin alone, and pre-treatment with melatonin increased LC3-II accumulation in AGE-induced EPCs (Fig. 2f). Western blotting showed that, compared with the AGEs-treated group, the LC3-II/LC3-I ratio was lower in the control group and significantly higher in the melatonin-pre-treated group. Treatment with melatonin alone also increased the LC3-II/LC3-I ratio compared with the control group, consistent with the fluorescence results, suggesting that melatonin promotes autophagy (Fig. 2e). The adaptor protein p62 (SQSTM1) connects LC3 protein to a ubiquitination substrate and can be incorporated into the complete phagosome by final autophagic lysosomal degradation, marking the completion of autophagic flux, and the accumulation of p62 indicates disrupted autophagic degradation^24^. In our study, Western blotting indicated that AGEs simultaneously increased p62 expression in EPCs. The fluorescence intensity and corresponding quantification showed that LC3 and p62 expression increased simultaneously with treatment with AGEs, suggesting that the initial accumulation of LC3 and autophagosomes after treatment with AGEs likely reflects a decrease in autophagy flux (Fig. 2e–h). In contrast, treatment with melatonin alone decreased the protein level of p62 compared with the control group, and melatonin pre-treatment attenuated p62 accumulation in AGE-induced EPCs. We also investigated the levels of lysosome-associated membrane protein 2 (LAMP2), a marker of lysosomal membranes that represents lysosomal function, to confirm whether lysosomal dysfunction contributed to the disruption of autophagy flux^25^. The levels of LAMP2 declined after treatment with AGEs, whereas melatonin treatment reversed these changes (Fig. 2e, g). This finding provides evidence that melatonin promotes autophagy flux and protects against lysosomal dysfunction in AGE-treated EPCs. We also detected activation of PINK1 and Parkin, a canonical mechanism for mitophagy regulation in most mammals^26^, in EPCs treated with AGEs and melatonin. The expression of both PINK1 and Parkin increased after stimulation with AGEs, whereas melatonin pre-treatment showed no significant effects on their expression (Fig. S2).

### The anti-apoptotic effect of melatonin is related to autophagy flux in AGE-treated EPCs

**Fig. 3:**
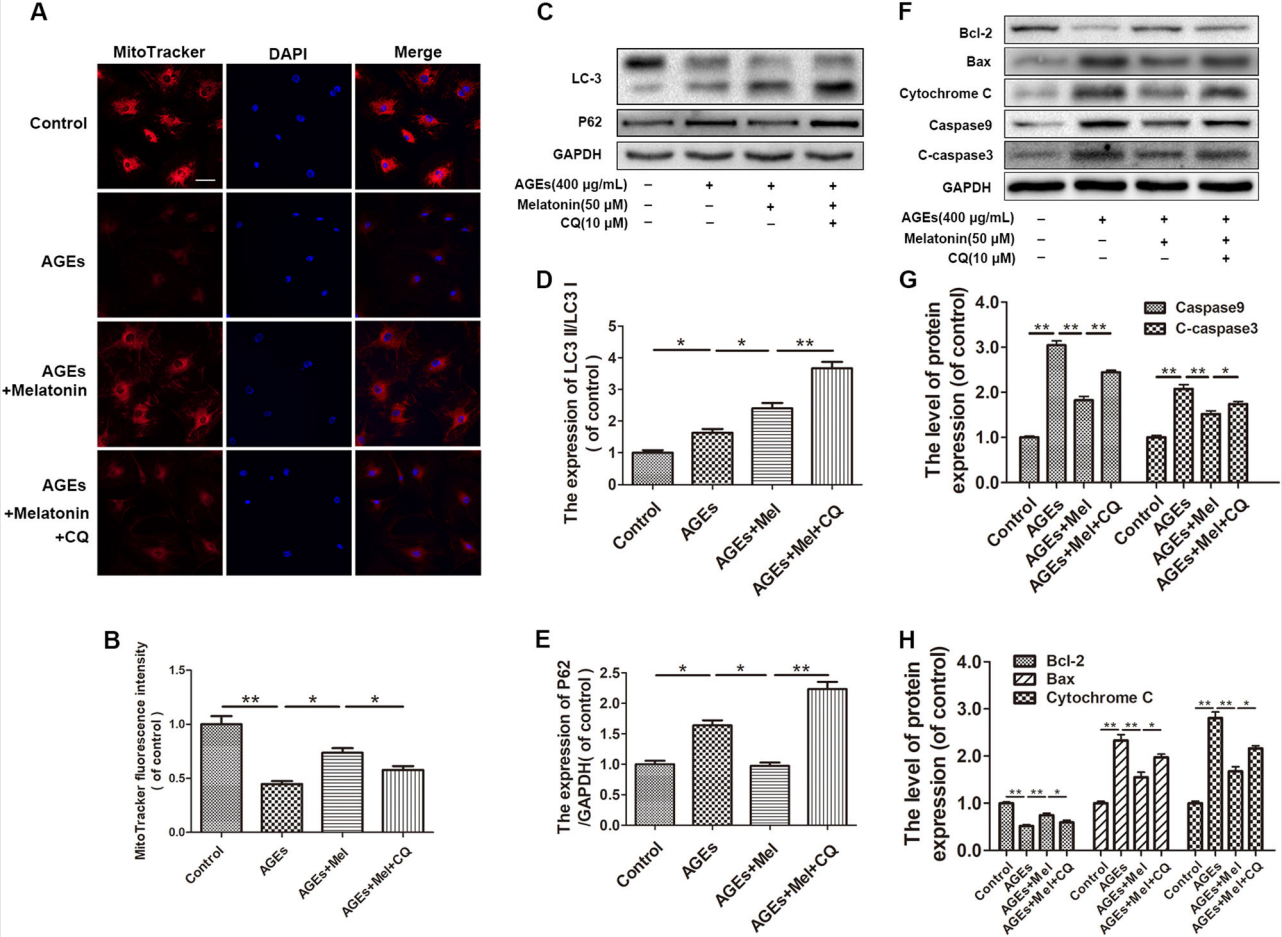
The anti-apoptotic effect of melatonin is related to autophagy flux in AGE-treated EPCs. EPCS were untreated or treated with AGEs (400 μg/mL) alone, melatonin (50 μM) + AGEs (400 μg/mL), or AGEs (400 μg/mL) + melatonin (50 μM) combined with chloroquine (CQ; 10 μM). **a**, **b** Mitochondrial membrane potential was detected using MitoTracker Red CMXRos, and nuclei were stained with Hoechst. Scale bar, 25 μm. **c**,**e** Protein expression levels of LC3, p62, and GAPDH in EPCs of each group treated as described above. **f**, **h** Protein expression levels of Bcl-2, Bax, cytochrome c, caspase-9, cleaved caspase-3, and GAPDH in EPCs of each group treated as described above. Data are presented as the mean ± SEM. Significant differences between the treatment and control groups are indicated as **P < 0.01 or *P < 0.05. *n* = 3

To investigate whether autophagy is involved in the melatonin-induced protective effect against apoptosis in EPCs, the cells were pre-treated with the autophagy inhibitor CQ. The membrane potential-dependent MitoTracker was utilized to assess MMP in EPCs. We found a significant reduction of red fluorescence in AGE-treated cells and melatonin markedly attenuated this decrease in fluorescence, whereas the CQ-pre-treated group showed the lowest levels, indicating that the activation of autophagy protects cells against apoptosis (Fig. 3a, b). Moreover, the Western blotting results also showed that CQ pre-treatment blocked autophagy flux (Fig. 3c, d) and reversed the effect of melatonin on decreasing apoptosis in EPCs (*P* < 0.01) (Fig. 3e–h). Thus, the activation of autophagy flux plays an important role in the anti-apoptotic effect of melatonin on AGE-treated EPCs.

### Melatonin improves the function of AGE-treated EPCs by promoting autophagy flux

**Fig. 4:**
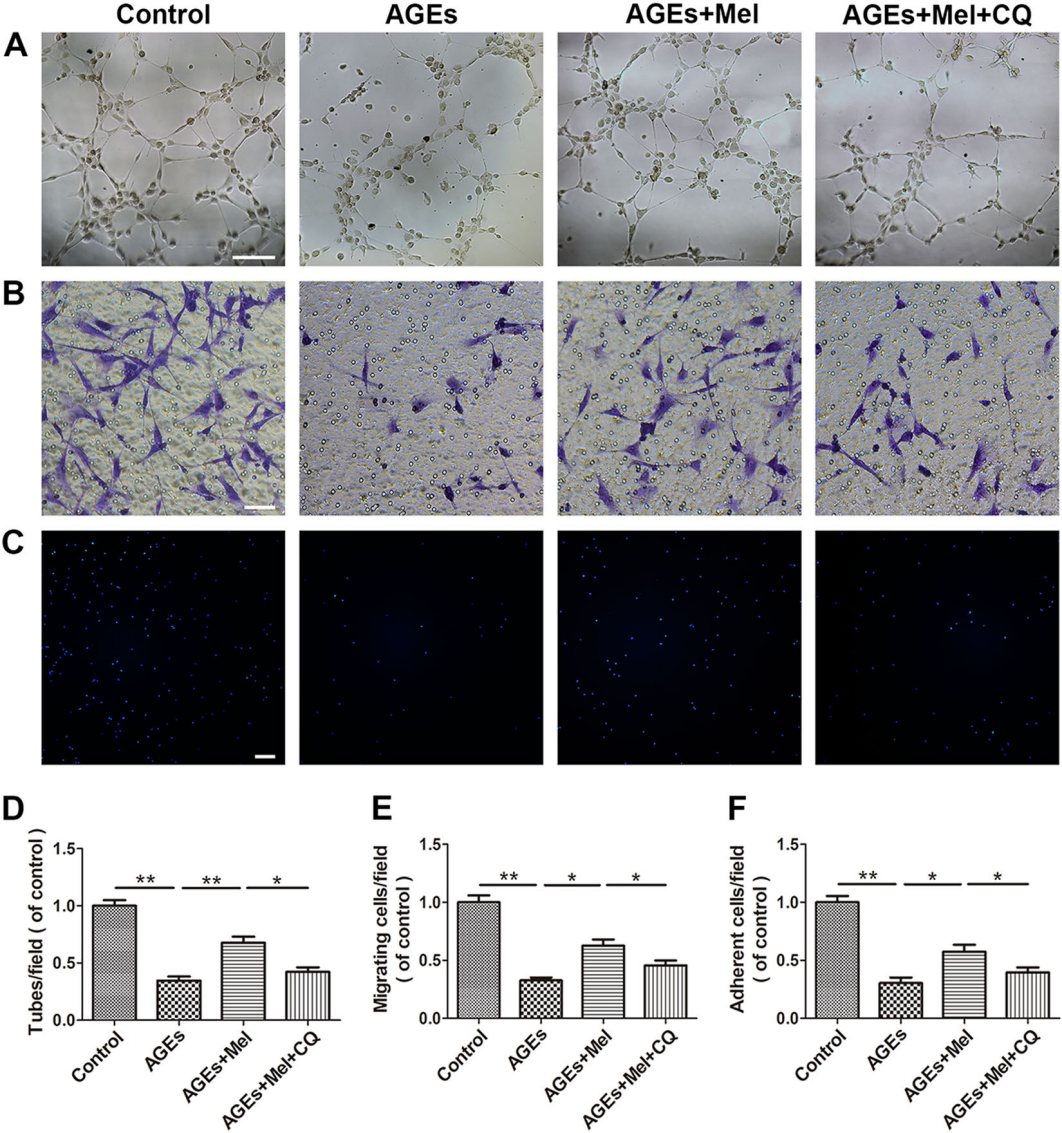
Melatonin improves the function of AGE-treated EPCs by promoting autophagy flux. **a**, **d** A tube formation assay was used to investigate the effect of melatonin on EPC neovascularization. Scale bar, 50 μm. **b**, **e** A transwell migration assay was used to study the effects of melatonin on EPC migration. Scale bar, 50 μm. **c**, **f** An adhesion assay was performed to determine the effects of melatonin on adhesion in EPCs. Scale bar, 100 μm. Data are presented as the mean ± SEM. Significant differences between the treatment and control groups are indicated as **P < 0.01 or *P < 0.05. *n* = 3

The efficacy of melatonin treatment on tube formation, migration, and adhesion in AGE-treated EPCs was investigated. First, a tube formation assay was used to investigate the effect of melatonin on EPC neovascularization. Representative photomicrographs showing the number of capillary-like structures in treated EPCs are shown in Fig. 4a. Treatment with AGEs significantly reduced the number of capillary-like structures (*P* *<* 0.05) (Fig. 4d). Compared to the AGE-treated group, melatonin effectively protected AGE-treated EPCs, with a significantly increased number of capillary-like structures, whereas co-treatment with CQ attenuated the efficacy of melatonin. Second, to study the effects of melatonin on EPC migration, transwell migration assays were carried out, and the migratory cell numbers of hematoxylin-stained basolateral membranes were counted (Fig. 4b). AGEs significantly suppressed the migration of EPCs by 64% compared with the control group. Compared with the AGE-treated group, the migration ability of cells in the melatonin group was significantly improved, and CQ inhibited the increase in migration ability conferred by melatonin (Fig. 4e). Finally, to investigate the effects of melatonin on EPC adhesion, an in vitro adhesion assay was performed to measure the adhesion of EPCs to fibronectin-coated culture plates (Fig. 4c). The number of adherent cells decreased after treatment with AGEs, and melatonin effectively protected the adherent ability of AGE-treated EPCs, which was weakened by the autophagy inhibitor CQ (Fig. 4f).

### Melatonin promotes autophagy flux by activating the AMPK/mTOR pathway in AGE-treated EPCs

**Fig. 5:**
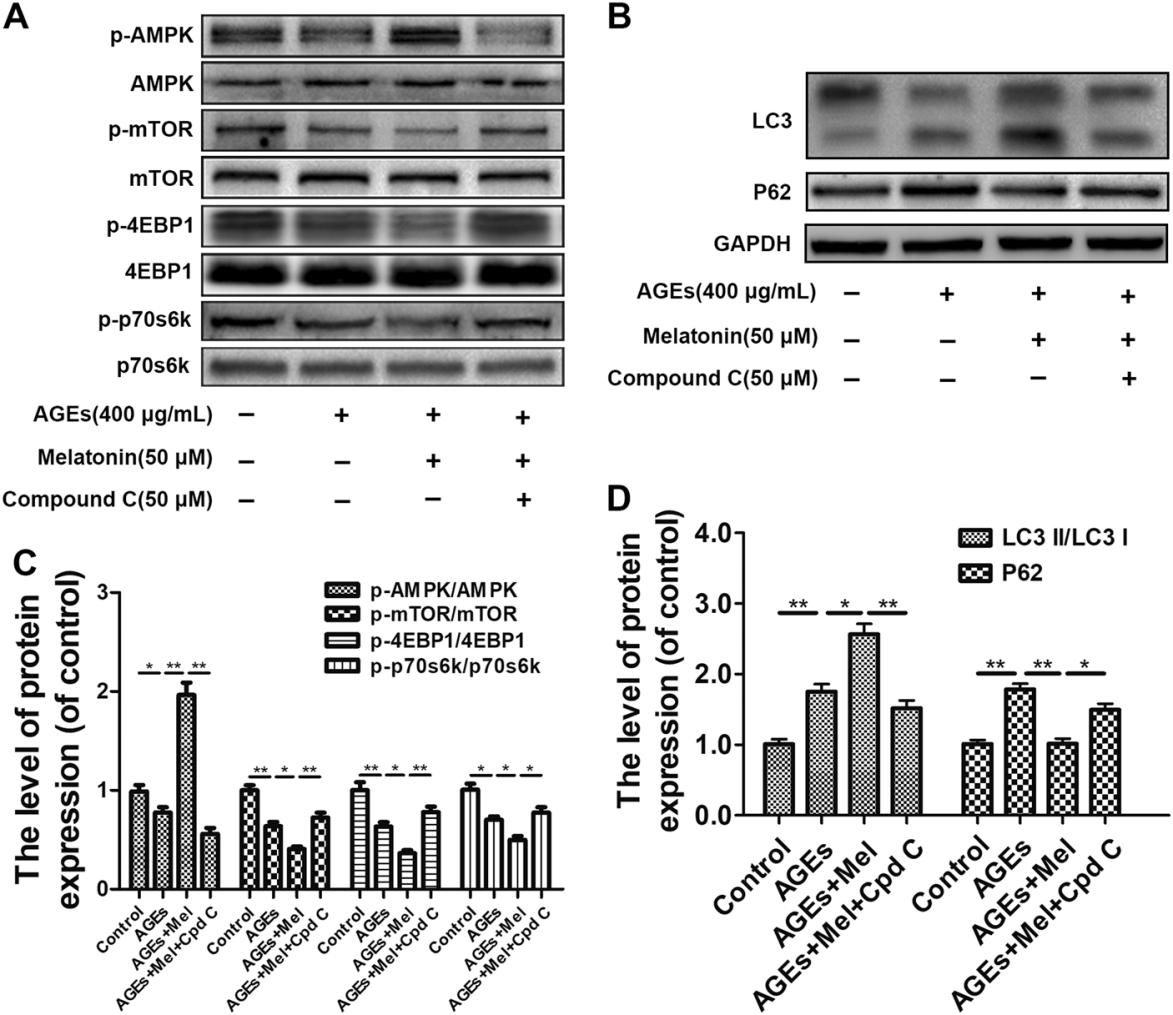
Melatonin promotes autophagy flux by activating the AMPK/mTOR pathway in AGE-treated EPCs. EPCs were pre-treated with 50 μM melatonin and 50 μM Compound C (Cpd C) for 2 h. Next, 400 μg/mL AGEs were added for an additional 6 h. **a**, **c** Representative Western blots and quantification data of p-AMPK/AMPK, p-mTOR/mTOR, p-p70s6k/p70s6k, and p-4EBP1/4EBP1 of each group treated with AGEs, melatonin, and Cpd C. **b**, **d** Representative Western blots and quantification data of LC3 and P62 of each group treated with AGEs, melatonin and Cpd C. Data are presented as the mean ± SEM. Significant differences between the treatment and control groups are indicated as **P < 0.01 or *P < 0.05. *n* = 3

To ascertain the mechanism underlying the observed effects of melatonin treatment, we explored whether the AMPK/mTOR pathway is involved in melatonin-stimulated autophagy flux. Western blotting was performed to investigate p-AMPK/AMPK, p-mTOR/mTOR, p-p70s6k/p70s6k, and p-4EBP1/4EBP1 expression in EPCs co-treated with melatonin and AGEs. Pre-treatment of the EPCs with AGEs had little effect on AMPK expression, but significantly decreased p-AMPK protein expression compared with control cells, whereas melatonin treatment significantly increased p-AMPK protein expression (Fig. 5a). Cpd C, an AMPK inhibitor, was also used to treat EPCs to confirm the role of the AMPK/mTOR pathway. Melatonin-induced autophagic responses in AGE-induced EPCs were attenuated when Cpd C suppressed p-AMPK activity (Fig. 5a, c). The ratios of p-mTOR/mTOR, p-p70s6k/p70s6k, and p-4EBP1/4EBP1 were enhanced by treatment with Cpd C, indicating that AMPK activation is required for autophagy in melatonin-treated EPCs (Fig. 5a–d). Taken together, these results suggest that melatonin activates the AMPK/mTOR pathway to promote autophagy after AGE treatment in EPCs.

### Melatonin promotes wound healing in diabetic mice

**Fig. 6:**
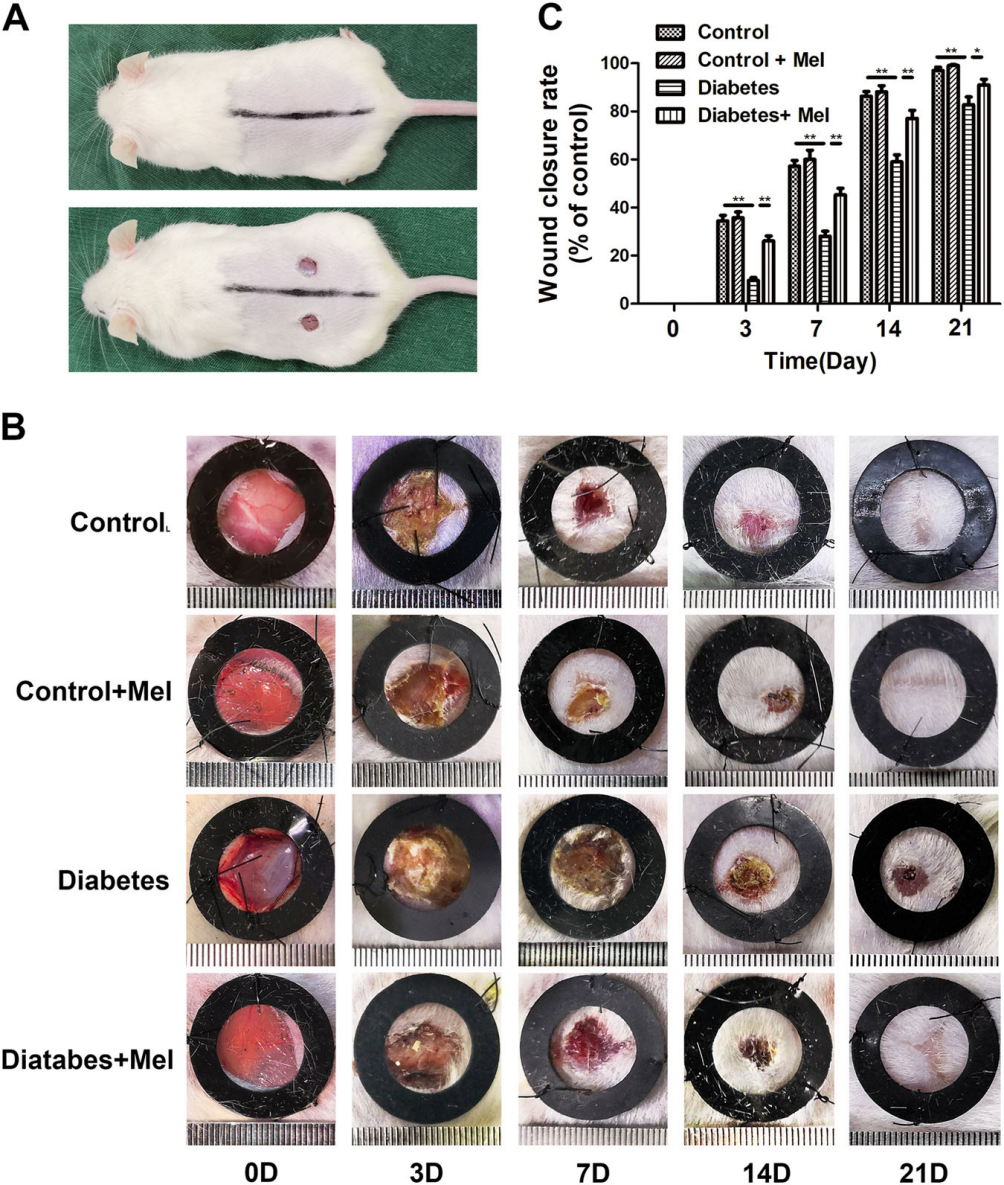
Melatonin accelerates wound closure. **a** Two rounds of full-thickness dermal wounds were made on both sides of the dorsal trunk. **b** Representative images of wound closure are shown in the control, control + melatonin, diabetes, and diabetes + melatonin groups on days 0, 3, 7, 14, and 21. **c** Wound closure rates of the four groups at the indicated times. Data are presented as the mean ± SEM. Significant differences between the treatment and control groups are indicated as **P < 0.01 or *P < 0.05. *n* = 6

**Fig. 7:**
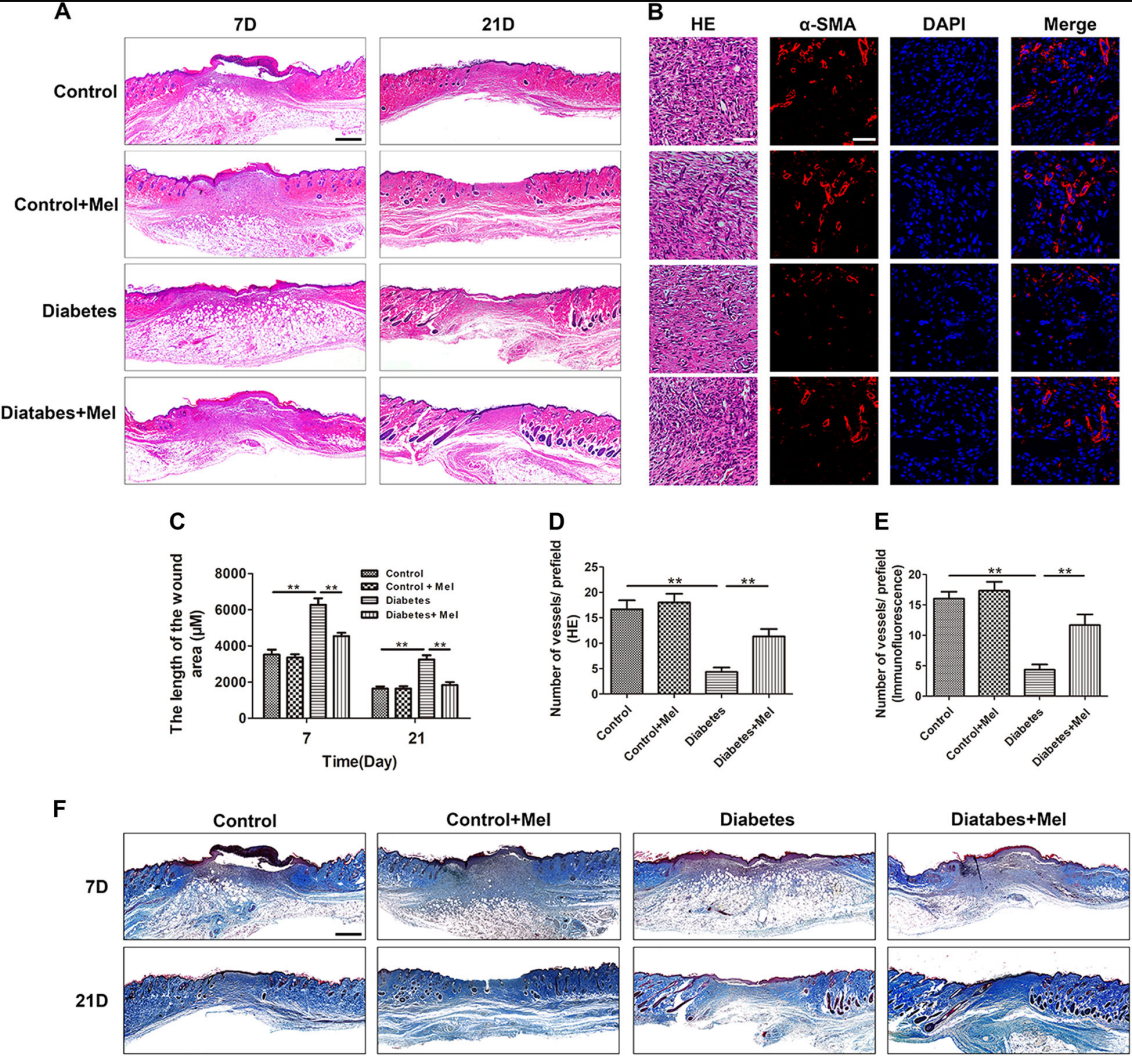
Melatonin promotes wound healing. **a** Hematoxylin and eosin (H&E)-stained images of wound healing in different treatment groups on days 7 and 21. Scale bar, 1000 μm. **b**, **c** New blood vessels were observed by α-SMA and H&E staining in enlarged images on day 7. Scale bar, 1000 μm. **d** Wound lengths in different treatment groups on days 7 and 21. **e**, **f** Quantitative analysis of the number of vessels per field on day 7 in H&E-stained and α-SMA-stained enlarged images. Scale bar, 100 μm. **g** Masson’s trichrome staining of wound healing in different treatment groups on days 7 and 21. Scale bar, 1000 μm. Data are presented as the mean ± SEM. Significant differences between the treatment and control groups are indicated as **P < 0.01

The surgical procedure is shown in Fig. 6a, and changes in body weight and blood glucose level are shown in Figure S3. The analytical results revealed that the wound closure rate was significantly lower in the control group than in the diabetes group on days 3, 7, 14, and 21, whereas there was no significant difference between the control group and the control + melatonin group (Fig. 6b, c). However, wound healing was significantly promoted in the diabetes + melatonin group compared with the untreated diabetes group on days 3, 7, 14, and 21 (Fig. 6b, c). As shown by the H&E staining, the length of the wound area in the diabetes + melatonin group on days 7 and 21 was significantly smaller than that in the diabetes group, although non-diabetic mice had the best wound healing (Fig. 7a, d). The enlarged images of H&E staining and α-SMA staining show significantly fewer capillaries in the wound bed in diabetic mice compared to non-diabetic mice, whereas treatment with melatonin increased the number of capillaries in diabetic mice (Fig. 7b, c, e, f). Collagen deposition is a critical factor in the proliferative phase of wound healing. Masson’s trichrome staining showed that the diabetes + melatonin group exhibited greater collagen deposition than the diabetes group on days 7 and 21 (Fig. 7g). Taken together, these data indicate that melatonin promotes wound healing in diabetic mice.

## Discussion

Increasing evidence shows that the accumulation of AGEs is one of the main mechanisms responsible for vascular damage in patients with diabetes, playing an important role in the pathogenesis of diabetic skin tissues and the impairment of diabetic wound healing^27^. Mitochondrial biogenesis plays an essential role in cellular adaptation and repair, whereas several studies have indicated that AGEs induce mitochondrial dysfunction that leads to mitochondrial-dependent apoptosis in cells^28^. In our study, AGEs caused mitochondrial dysfunction in EPCs and led to apoptosis. Meanwhile, the functions of EPCs, including formation, migration, and adhesion, were impaired under AGE treatment. After pre-treatment with melatonin, compared to the AGE-treated group, the expression of Bax and cleaved caspase-3 decreased, whereas Bcl-2 increased, suggesting that the mitochondrial pathway may be involved in the anti-apoptotic effect of melatonin. Melatonin also improved EPC functions in AGE-treated EPCs.

Numerous studies have shown that moderating autophagy has protective effects against various pathologies^29,30^. As autophagy research has developed, increasing attention has focused on intact autophagy flux. Several recent studies have shown that excessive numbers of autophagosomes do not necessarily correlate with increased autophagic activity or flux, which may be caused by the stimulation of autophagy or by the reduced degradation of autophagy^31^. Chen et al.^32^ reported that AGEs increased the ratio of LC3-II/I but had no obvious effect on p62 protein expression in HUVECs. Li et al.^33^ demonstrated that AGEs dramatically increased the expression of LC3-II/I and p62 in HUVECs. In our study, we observed a similar phenomenon in EPCs, as AGEs increased the ratio of LC3-II to LC3-I, while p62 was also accumulated, suggesting the impairment of autophagic flux rather than the activation of autophagy. Lysosomes play a crucial role in autophagic flux, which is also called the lysosome-dependent pathway, and are the key organelles responsible for successful autophagic degradation. To further examine the disrupted autophagy–lysosome pathway, we examined the levels of LAMP2, a lysosomal membrane marker, and found that they decreased after treatment with AGEs. Therefore, it is necessary to restore intact autophagy flux in AGE-treated EPCs.

**Fig. 8:**
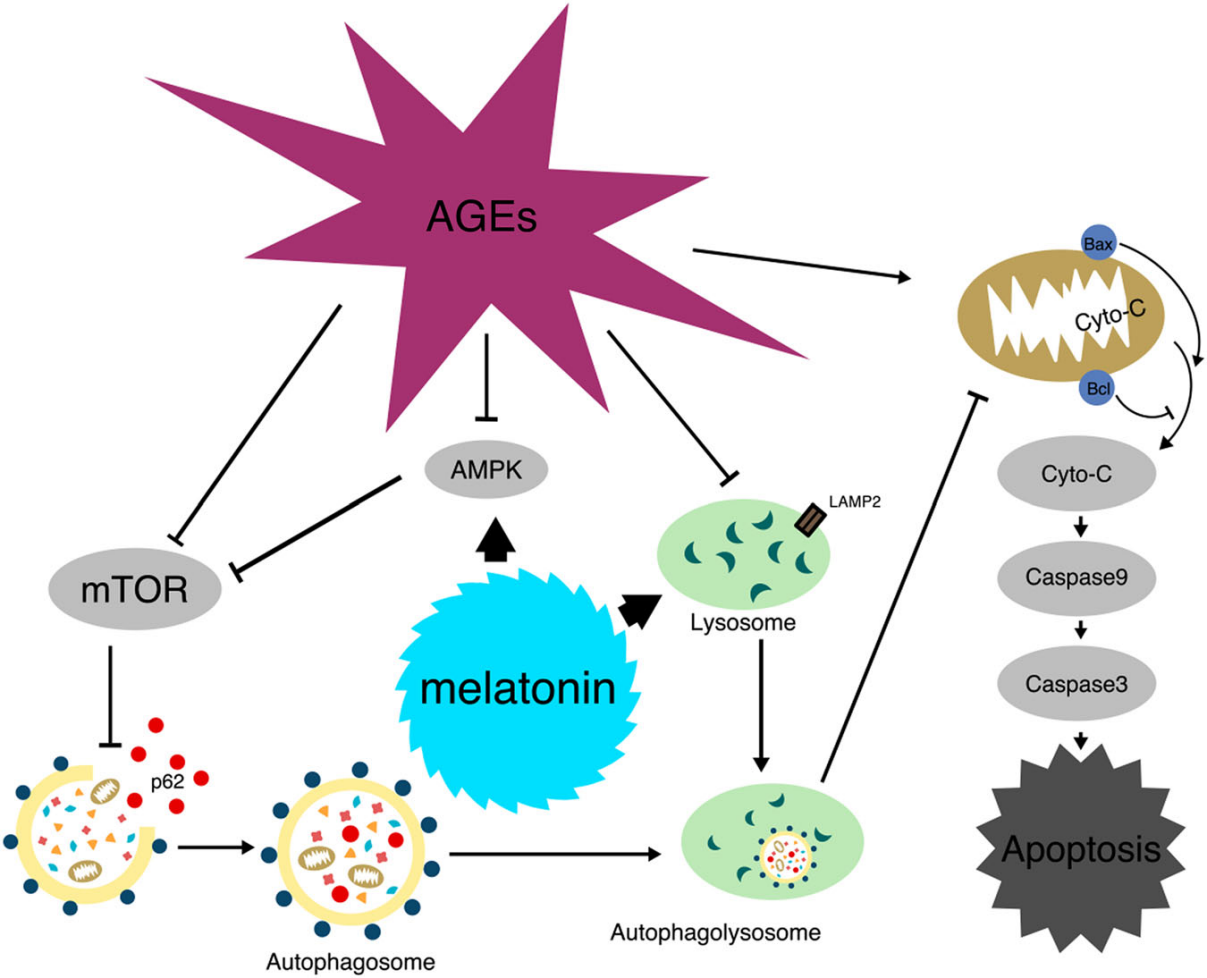
Schematic model of the signaling mechanisms underlying the beneficial effects of melatonin on AGE-induced apoptosis of EPCs

We evaluated the effects of melatonin on autophagy flux in EPCs after treatment with AGEs. Kongsuphol et al.^17^ reported that melatonin suppressed autophagy in an SK-N-SH dopaminergic cell line, and Yoo and Jeung^18^ indicated decreased autophagy in melatonin-treated rat pituitary GH3 cells. However, other studies have shown that melatonin may induce autophagy and thereby protect cells against harmful stimulation^20,21^. Our results showed that the ratio of LC3-II to LC3-I increased, whereas p62 expression decreased significantly, which indicates that melatonin increases the formation of autophagosomes in AGE-treated EPCs. In addition, melatonin recovered the expression of LAMP2, which decreased after AGEs treatment. Similarly, Li et al^34^. found that melatonin preserved lysosomal protease activity, including increasing LAMP2 levels, by maintaining lysosomal pH levels, and restored cell viability in cadmium-treated cells. These results indicate that melatonin also repairs the function of lysosomes, which may contribute to the restoration of the autophagy–lysosome pathway (Fig. 8). Furthermore, the expression levels of both PINK1 and Parkin increased after stimulation with AGEs, whereas pre-treatment with melatonin did not cause a significant change. This result indicates that mitophagy may not play a key role in the protective effect of melatonin. To reveal the relationship between autophagic flux and mitochondrial dysfunction, we applied CQ, an inhibitor of autophagosome degradation. Inhibiting autophagic flux significantly counteracted melatonin-induced improvements in cell function. This result suggests that the protective effect of melatonin on mitochondrial function is mainly mediated by intact autophagy flux.

Melatonin is an activator of AMPK signaling^35^, and activating p-AMPK can inhibit the mTOR signaling pathway, which is a classic autophagy activation pathway. We demonstrated that melatonin promoted p-AMPK and inhibited the mTOR signaling pathway, including p-mTOR/mTOR, p-P70/P70, and p-4EBP1/4EBP1, to promote the activation of autophagy. Pre-treatment with an AMPK inhibitor, Cpd C, significantly abolished the effects of melatonin, as identified by the downregulation of p-AMPK, upregulation of p-mTOR, and decreased autophagosome formation. Therefore, the protective effects of melatonin on AGE-treated EPCs are AMPK-dependent.

To verify the therapeutic effects of melatonin in vivo, a diabetic wound healing model was established and the wound healing process was evaluated after 3, 7, 14, and 21 days of successful modeling. Diabetic mice showed delayed wound healing compared with non-diabetic mice. Following the administration of melatonin, no significant differences were found between the control group and the control + melatonin group, consistent with the results described by Lee et al^36^. However, melatonin sped up the healing process in diabetic mice, and more capillaries were observed in mice in the diabetes + melatonin group than in the diabetes group. The strength and appearance of scars in the diabetes + melatonin group, which had much more collagen deposition, also appeared better than those in the diabetes group.

However, the in-depth mechanism by which melatonin repairs lysosomal function is still unclear. Although changes in the internal lysosomal pH are likely involved, further investigations are still needed. Moreover, our in vivo experiment did not provide the most direct evidence of the protective role of EPCs in wound healing. Interestingly, several studies have reported that EPCs mobilize more to the wound for neovascularization by effective treatment^37,38^, which supports their protective role in wound healing.

In conclusion, this study showed that melatonin treatment induces autophagy flux in an AMPK-dependent manner in AGE-treated EPCs, which protects mitochondria and confers anti-apoptotic and anti-dysfunction effects. The in vivo experiment also indicated that melatonin promotes wound healing in diabetic mice. Thus, our study revealed a novel working mechanism of action for melatonin in diabetic wound healing.

## Electronic supplementary material

Supplemental Figure Legends

Supplemental Figure 1

Supplemental Figure 2

Supplemental Figure 3
