# Supplementary material for: Melatonin protects endothelial progenitor cells against AGE-induced apoptosis via autophagy flux stimulation and promotes wound healing in diabetic mice
Source: Exp Mol Med. 2018 Nov 21;50(11):154. doi: 10.1038/s12276-018-0177-z (PMC6249246; doi:10.1038/s12276-018-0177-z)
Supplement: Supplementary file 1 — Supplemental Figure Legends [file 12276_2018_177_MOESM1_ESM.doc]

**Supplemental Figure Legends**

**Supplemental Figure 1.** The morphology and characterization of bone marrow-derived EPCs. (A) The morphology of EPCs in different growth periods. Scale bar, 50 μm. (B) Most early EPCs were shown to endocytose, simultaneously, DiI-acLDL (red) and FITC-labeled UEA-1 lectin (green). Scale bar, 50 μm. (C) Double immunofluorescence of endothelial markers CD31 (red) and KDR (green) in EPCs. Scale bar, 50 μm. (D) Representative image of tubuli-like structures formed on Matrigel by late EPCs. Scale bar, 50 μm.

**Supplemental Figure 2.** The effect of melatonin on the PINK1/Parkin pathway in AGEs-treated EPCs. EPCs were pre-treated with 50 μM melatonin for 2 h, and then 400 μg/mL AGEs were added for an additional 24 h. The protein expression levels of PINK1 and Parkin were visualized by Western blot (A) and quantified (B). Data are presented as mean ± SEM. Significant differences between the treatment and control groups are indicated as ***P* < 0.01 or **P* < 0.05. N.S. indicates *P* ≥ 0.05. n = 3.

**Supplemental Figure 3.** Changes in body weight (A) and blood glucose (B) of different groups of mice at the indicated days after injection. Data are presented as mean ± SD. Significant differences between the control group and the diabetes group are indicated as ***P* < 0.01 or **P* < 0.05. n = 6.
