# Supplementary figures and images for: Melatonin protects endothelial progenitor cells against AGE-induced apoptosis via autophagy flux stimulation and promotes wound healing in diabetic mice
Source: Exp Mol Med. 2018 Nov 21;50(11):154. doi: 10.1038/s12276-018-0177-z (PMC6249246; doi:10.1038/s12276-018-0177-z)

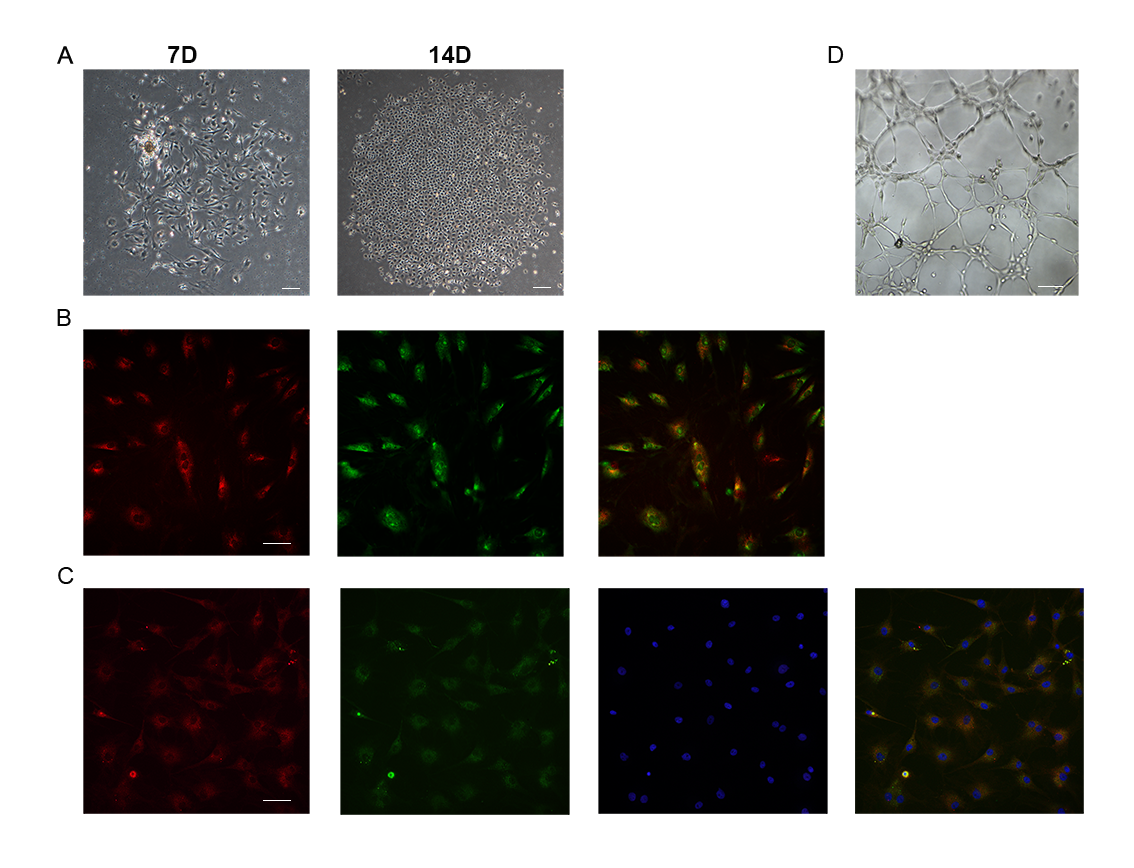

Supplement: Supplementary file 2 — Supplemental Figure 1 [file 12276_2018_177_MOESM2_ESM.tif]

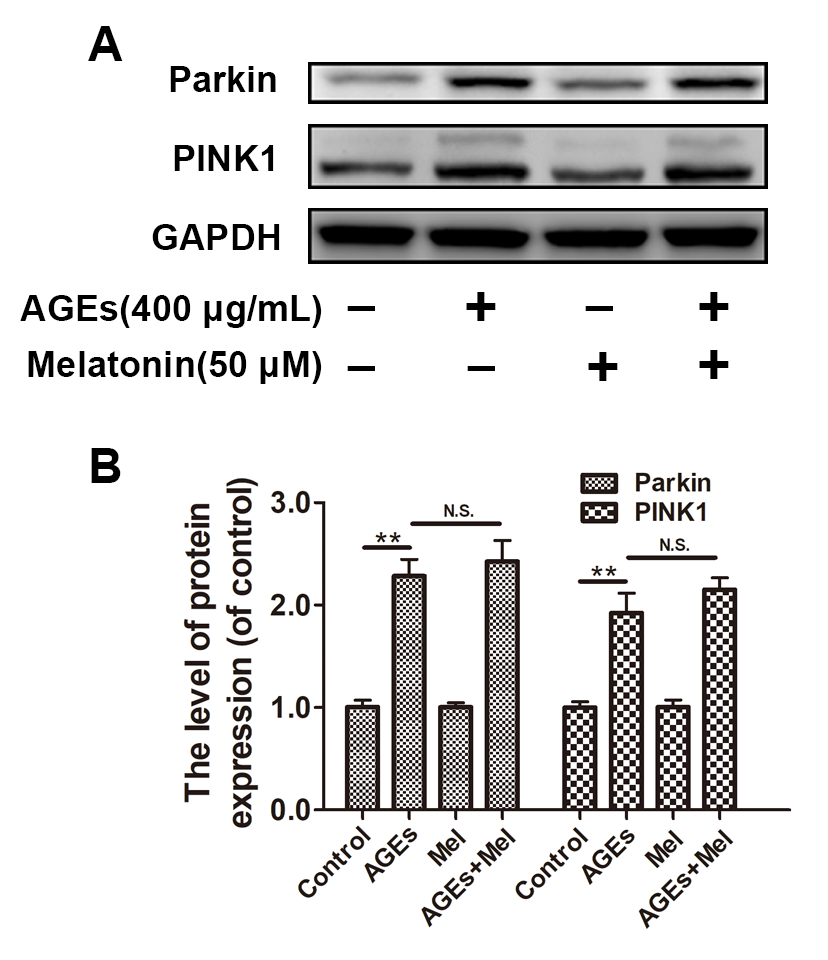

Supplement: Supplementary file 3 — Supplemental Figure 2 [file 12276_2018_177_MOESM3_ESM.tif]

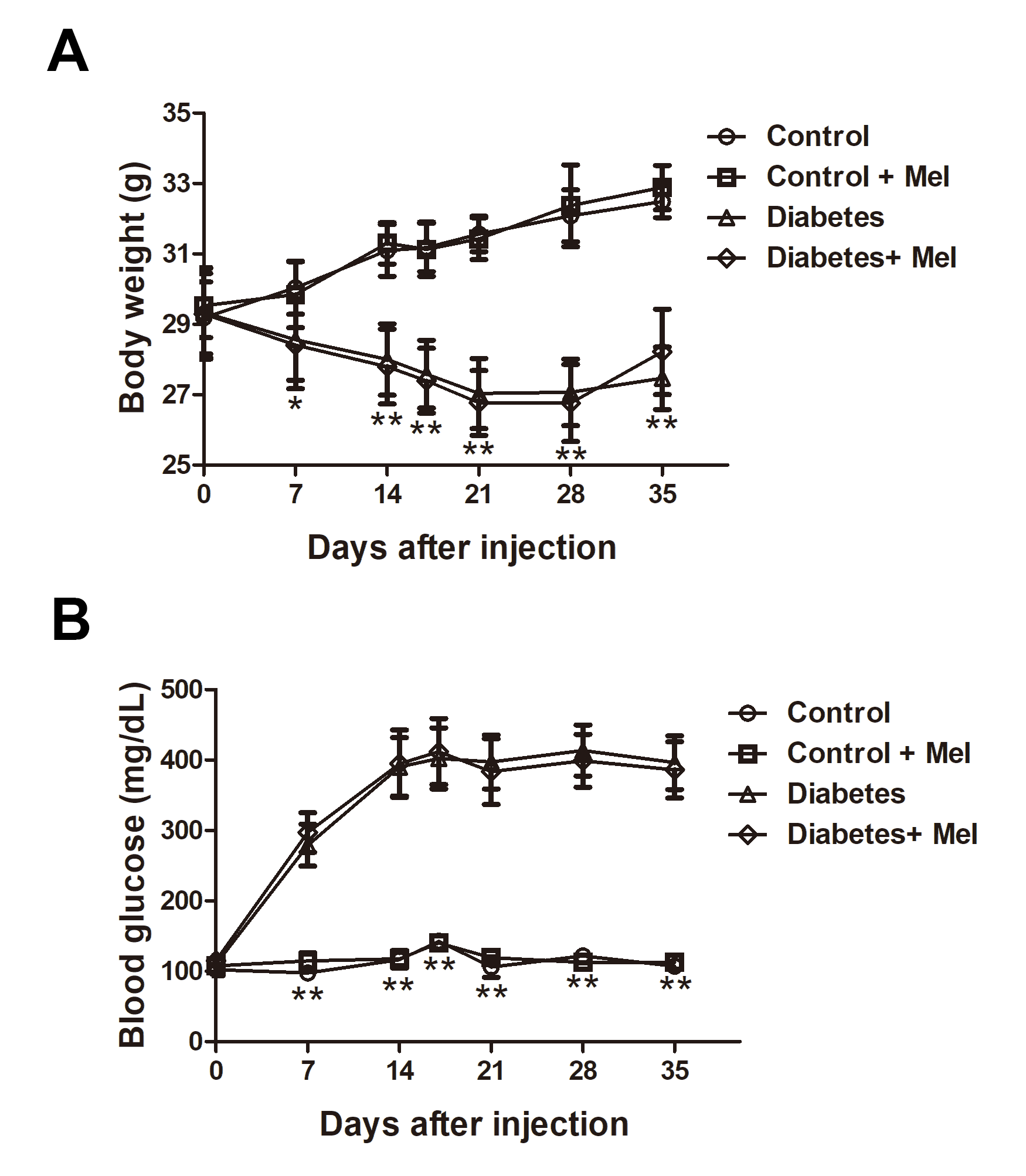

Supplement: Supplementary file 4 — Supplemental Figure 3 [file 12276_2018_177_MOESM4_ESM.tif]
